# Internal Occipital Crest Misalignment with Internal Occipital Protuberance: A Case Report of Posterior Cranial Fossa Anatomic Variations

**DOI:** 10.1155/2016/7575623

**Published:** 2016-08-25

**Authors:** Jae Ha Kim, Maha Ahmad

**Affiliations:** Department of Biomedical and Diagnostic Sciences, University of Detroit Mercy School of Dentistry, Detroit, MI, USA

## Abstract

During gross anatomy head and neck laboratory session, one dissection group observed an abnormal anatomic variation in the posterior cranial fossa of a 94-year-old male cadaver. The internal occipital crest was not aligned with internal occipital protuberance and groove for superior sagittal sinus. It seemed that the internal occipital protuberance was shifted significantly to the right side. As a result the skull was overly stretched in order to connect with the internal occipital ridge. These internal skull variations of occipital bone landmarks can influence the location of adjacent dural venous sinuses and possibly influence cerebrospinal fluid flow. Similar anatomical anomalies have been attributed to presence of hydrocephalus and abnormalities in cisterna magna.

## 1. Introduction

The posterior cranial fossa is posteriorly enclosed by the occipital bone. Internal occipital crest and internal occipital protuberance are prominent anatomical landmarks of the floor of the midsagittal plane of posterior cranial cavity. The internal occipital protuberance is identified as an elevated part of cruciform eminence which divides the occipital bone into four fossae: two upper cerebral fossae and two lower cerebellar fossae. The groove of transverse sinus separates the upper cerebral fossae from the lower cerebellar fossae. The groove for superior sagittal sinus is superiorly located to the protuberance. An elevated ridge that begins from the internal occipital protuberance down to the foreman magnum is the internal occipital crest, which also serves as attachment site for the falx cerebelli. Normally, the internal occipital crest, protuberance, and groove for superior sagittal sinus are aligned in a straight line. Occipital protuberance and internal occipital crest, as a group, are closely related to dural venous sinuses and binding of falx cerebri and falx cerebelli. Internal occipital crest traces the occipital sinus to the confluence of the sinus, located next to the internal occipital protuberance. These sinuses are located within layers of dura maters. Internal occipital protuberance is an important landmark where confluences of the transverse, occipital, and superior sagittal sinuses meet and falx cerebri and falx cerebelli folds attach.

## 2. Case Report

**Figure 1:**
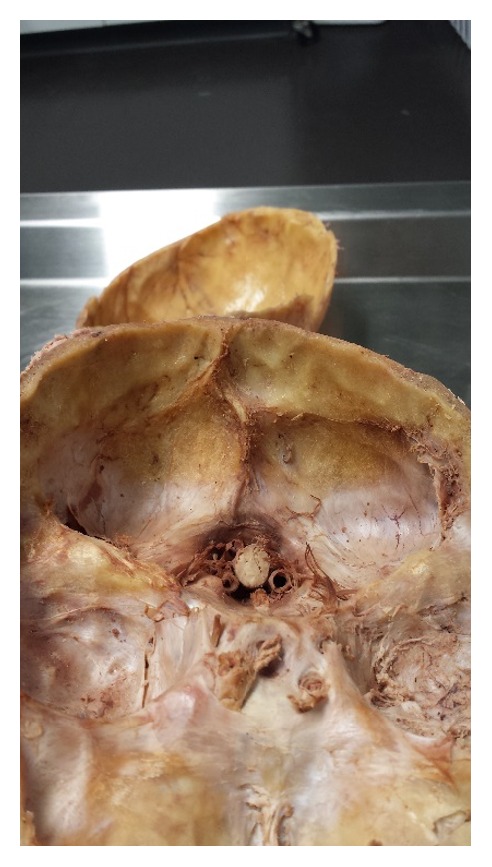
Dissection of the skull by making horizontal cuts to expose internal bony landmarks.

**Figure 2:**
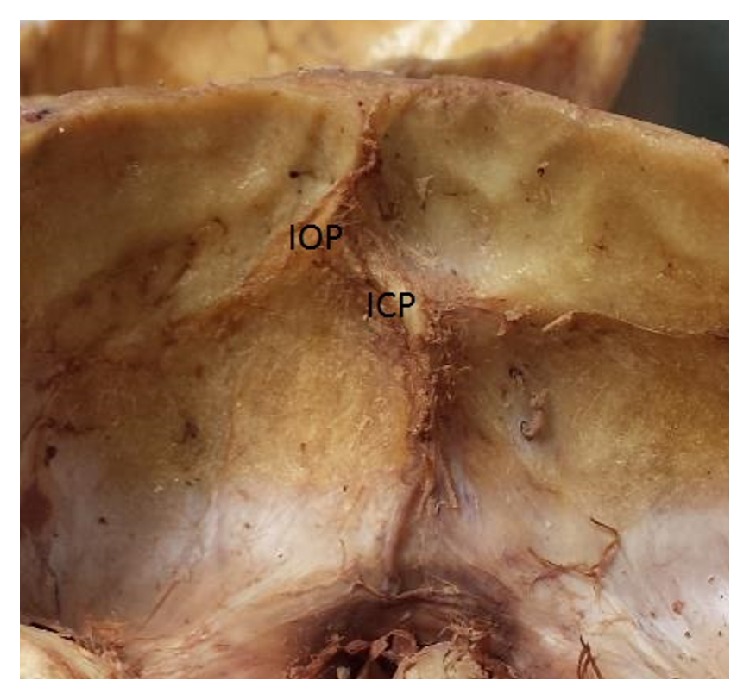
Frontal/superior view of the occipital bone and the misalignment of internal occipital crest (IOC) and internal occipital protuberance (IOP).

**Figure 3:**
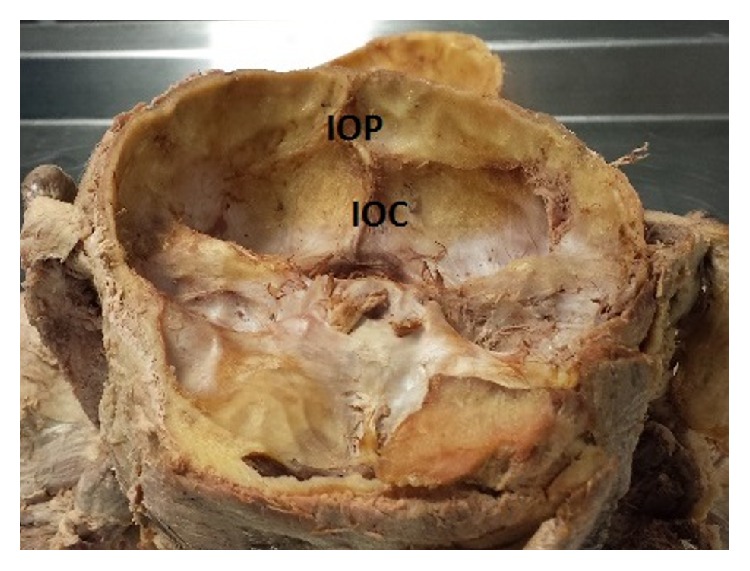
Enlarged view of internal occipital protuberance and internal occipital crest showing the misalignment of internal occipital crest (IOC) and internal occipital protuberance (IOP).

**Figure 4:**
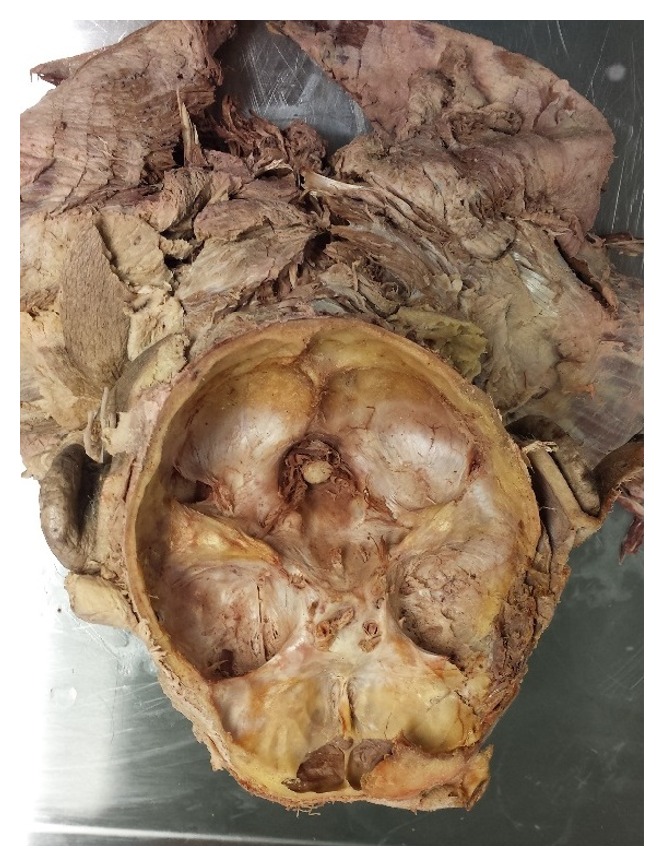
Superior view showing the reduction of space of the right side due to the shift of internal occipital protuberance to the right compared to the left side of the skull.

An obvious misalignment of the internal occipital protuberance and internal occipital crest was observed during a head and neck laboratory session for dental school students. It was noticed in a 94-year-old cadaver who died from malignancy of the prostate, ischemic heart disease, and cirrhosis of liver. To reveal the internal bony landmarks, horizontal cuts were made through temporal bone, frontal bone, and occipital bone of the skull using a schissel and a hammer (Figure 1). The internal occipital protuberance and groove for superior sagittal sinus are shifted to the right of the skull, as shown in Figures 2 and 3. However, the internal occipital crest was normally positioned in the inferior center of the occipital bone and foramen magnum. Groove for superior sagittal sinus and internal occipital protuberance was moved significantly to the right of the skull. The students who participated in the dissection found that the right cerebellar hemisphere was smaller compared to the left cerebellar hemisphere. As seen in Figures 2 and 4, there is a smaller cavity for right occipital lobe and right cerebellar hemisphere. The possibility of hydrocephalus and cisterns magna problems [1, 2] was suspected to be associated with inflammation/abnormality of the brain and was mentioned due to the shift of the internal occipital protuberance to the right, but the dissection group did not recognize any abnormality/inflammation of the brain.

**Figure 5:**
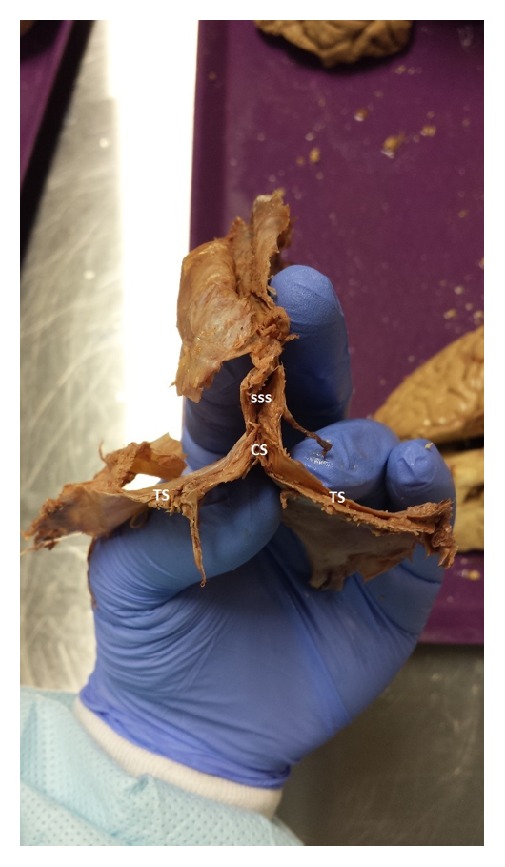
Dura mater with superior sagittal sinus (SSS), two transverse sinuses (TS), and confluence of sinus (CS) joining the three sinuses.

**Figure 6:**
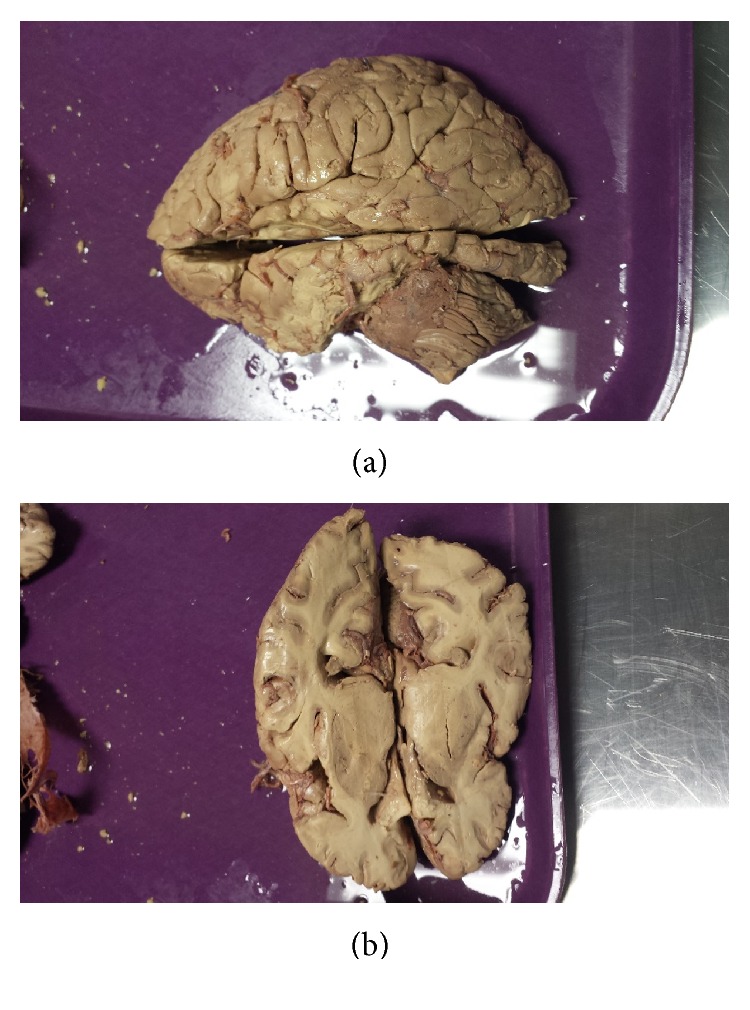
Sagittal dissection of the brain (a) and horizontal dissection of the brain (b). This figure shows absence of inflammation of the brain and the size differences between right and left cerebrum.

Figure 5 shows that there were no abnormalities associated with dura mater, superior sagittal sinus, transverse sinus, and confluence of the sinus. Two different dissections of the brain are shown in Figure 6. Both sagittal and horizontal dissections made on the brain were unremarkable. However, there was a noticeable size difference between right and left cerebral hemispheres. As Figure 6 horizontal dissection shows, the posterior regions of left and right occipital lobe do not align with each other. This is a possible abnormality caused by the lateral shift of internal occipital protuberance to the right, causing the right occipital lobe to move downwards. As a result, right occipital lobe is larger compared to the left occipital lobe. Also the right cerebellum is reduced in size compared to the left cerebellum perhaps due to the larger right occipital lobe.

## 3. Discussion

Internal occipital crest misalignment with internal occipital protuberance has been reported in the literature as a byproduct of select brain pathologies. According to a case report by Pozzati et al., 1979 [2], there was an incidence of hydrocephalus associated with anomalous internal occipital crest. In this case report, we could not observe any evidence of major inflammation and/or brain abnormalities. Therefore, the possibility for hydrocephalus and cisterna magna problems [1] due to the shift of internal occipital protuberance and misalignment with internal occipital crest remains uncertain. However, another possibility is the presence of greater protrusion of the occipital pole of the hemisphere or what is defined as “occipital petalia” [3], which is often related to occipital skull asymmetries [4]. Petalias are believed to be associated with increased hemispheric specialization of function [3] and may occur as a result of right and left cerebral hemispheres spatial displacement [5]. Left occipital petalias were found to be more frequent than the right occipital petalias in healthy young adults [5]. This might explain the larger left occipital pole found in this case report.

In conclusion, further morphometric studies and clinical analysis are needed in order to reveal whether these skull/brain asymmetries are normal neuroanatomical variations or caused by certain brain pathologies.
